# Communication of Illness Severity to Parents in the Neonatal Intensive Care Unit: A Scoping Review of Communication Characteristics and Prognostic Framing

**DOI:** 10.3390/children13081006

**Published:** 2026-07-29

**Authors:** Yasmine Tremblay, Kyra McKinnon, Jeneesha Dhaliwal, Sandesh Shivananda

**Affiliations:** 1 British Columbia Children’s Research Institute, University of British Columbia (UBC), Vancouver, BC V6H 3N1, Canada; kyra.mckinnon@cw.bc.ca; 2 Women’s Health Research Institute, British Columbia Women’s Hospital, Vancouver, BC V6H 3N1, Canada; jeneesha.dhaliwal@cw.bc.ca; 3 Division of Neonatology, University of British Columbia (UBC), Vancouver, BC V6H 3N1, Canada

**Keywords:** neonatal intensive care, illness severity, family-centred care, communication, parent, clinicians

## Abstract

**Highlights:**

**What are the main findings?**

- Communication about illness severity in the NICU was most often clinician-led and focused on providing biomedical information rather than supporting interactive discussion or shared decision-making.
- Few studies examined how communication changed across periods of clinical stability, deterioration, or recovery. The studies inconsistently described prognostic framing and parental engagement.

**What are the implications of the main findings?**

- Greater attention to illness trajectory and parental information needs may help improve communication practices in the NICU.
- Future research should better distinguish between informational communication and communication intended to support parental decision-making, particularly during changes in illness severity.

**Abstract:**

**Background/Objectives:** Communication about illness severity in the neonatal intensive care unit (NICU) occurs in settings of uncertainty and changing clinical status. Reviewing how the literature describes this communication may clarify current practices and identify gaps in how parents receive severity-related information. This scoping review aimed to describe how illness severity is communicated to parents in the NICU, including parental engagement, communication goals, informational content, framing, and communication across periods of clinical stability and deterioration. Methods: We conducted a scoping review using the Joanna Briggs Institute method and reported it according to PRISMA-ScR guidelines. MEDLINE (Ovid) was searched from 2010 to 2025. We included studies that reported empirical data on communication about infant clinical status or illness severity and included parental perspectives. A framework, informed by parental information behaviour and prognostic communication models, guided the data extraction. **Results:** We included 25 studies. Communication was most often clinician-led and often combined active and passive approaches. Communication primarily focused on information sharing rather than decision-making. Discussions were largely biomedical; however, several studies also included treatment- and care-related information. Studies inconsistently described prognostic communication and did not emphasize optimistic or pessimistic framing. Few studies examined communication across distinct phases of illness trajectory, such as clinical stability or deterioration. **Conclusions**: Illness severity communication in the NICU remains largely clinician-led, with limited attention to parental engagement and changes across the infant’s clinical course. Greater consideration of parental information needs, prognostic communication, and illness trajectory may support more responsive communication practices in NICU care.

## 1. Introduction

The neonatal intensive care unit (NICU) is a setting of clinical uncertainty, changing illness trajectories, and emotionally difficult decision-making [1,2]. Parents of critically ill infants must interpret complex medical information while coping with stress, fear, and prolonged hospitalization [3,4]. Communication about illness severity is therefore not limited to isolated conversations but occurs throughout the infant’s clinical course [1]. How information is communicated can influence parental understanding, trust, engagement, and involvement in care and decision-making [1,5,6,7].

Communication about illness severity includes discussion of the infant’s current condition, changes in clinical status, treatment plans, prognosis, and uncertainty [1,2]. These conversations may occur during bedside updates, family meetings, rounds, or urgent clinical events [3]. They may involve an explanation of physiologic parameters, treatment plans, and prognosis, and acknowledgement of uncertainty [8,9]. Presenting information optimistically, pessimistically, or in a balanced manner can shape parents’ interpretation of prognosis and expectations [10]. Communication may also serve different purposes, ranging from providing clinical updates to supporting shared decision-making [1,2].

Existing literature on NICU communication has largely focused on family-centred care, shared decision-making, parental experiences, and prognostic discussions in neonatal and pediatric critical care settings [1,2,3,4,5,6,7,8]. However, studies often examine communication broadly. They do not distinguish between information sharing and decision support, nor do they consider how communication changes across the infant’s clinical course [4]. Important aspects of illness severity communication, including parental engagement, framing, timing, communication modality, and informational content, remain poorly characterized and inconsistently reported in the existing literature [1].

Little information exists on how illness severity communication differs across NICU care phases, such as when a patient is stable or their condition worsens [2,4]. It is also unclear how parents participate in these discussions or how informational communication differs from communication intended to support decision-making. Examining these patterns may help identify gaps in current communication practices and areas for improvement in NICU care.

The aim of this scoping review was to describe how illness severity is communicated to parents of infants in the NICU, including parental engagement, communication goals, informational content, framing, and communication across periods of clinical stability and deterioration.

### Research Question

How is illness severity communicated to parents of infants in the NICU, with respect to parental engagement, communicative purpose, informational content, framing style, and the temporal context (including periods of stability and deterioration)?

## 2. Materials and Methods

### 2.1. Study Design

This scoping review followed the Joanna Briggs Institute (JBI) methodology for scoping reviews and used the PRISMA-ScR (Preferred Reporting Items for Systematic Reviews and Meta-Analyses extension for Scoping Reviews) checklist for reporting [11,12] (Supplementary Table S1). We chose a scoping review approach because the literature on illness severity communication in the NICU includes qualitative, quantitative, and mixed-method studies, as well as studies examining digital communication tools and physiologic displays [12,13]. This review describes how the literature represents illness severity communication and identifies key patterns and gaps in current communication practices. We did not prospectively publish a protocol for this scoping review.

### 2.2. Research Question Development

The review was initially focused on communication strategies involving real-time physiologic data and digital tools in the NICU. The original research question examined how these approaches influenced parental understanding, preferences, and satisfaction compared with standard communication methods. A PICO framework was used to guide question development [14].

During screening and data extraction, few studies directly compared digital or physiologic data-based communication tools with standard communication approaches. We therefore broadened the review’s scope to examine illness severity communication more generally, including parental engagement, communication goals, informational content, framing, and communication across the infant’s clinical course.

### 2.3. Search Strategy

We developed a search strategy in collaboration with a research librarian (PG) to identify literature on illness severity communication in the NICU. The search combined controlled vocabulary and keywords related to NICU parents and caregivers, illness severity and clinical status, and communication.

On 6 May 2025, we searched MEDLINE (Ovid) for studies published between 2010 and 2025. Search terms included concepts related to illness severity, prognosis, clinical deterioration, and provider–family communication. Supplementary Table S2 provides the full search strategy.

We selected MEDLINE because it broadly covers neonatal and clinical literature. Records were imported into Covidence (Veritas Health Innovation, Melbourne, Australia) for screening and data management. Reference lists of included studies were also reviewed to identify additional articles [13].

### 2.4. Eligibility Criteria

We included studies that reported empirical data on communication concerning infant clinical status, illness severity, physiologic stability, or medical complexity during NICU hospitalization and that also incorporated parental perspectives (e.g., preferences, understanding, satisfaction, or experiences). Qualitative, quantitative, and mixed-method studies were eligible. We also included studies that examined verbal, written, or digital communication approaches.

Studies were excluded if they focused exclusively on long-term prognosis or discharge counselling, included only clinician perspectives, were non-empirical (e.g., editorials or commentaries), or were not published in English.

### 2.5. Study Selection

We imported all retrieved citations into Covidence and removed duplicates before screening. Study selection was conducted in two stages: title and abstract screening, followed by full-text review.

Before screening began, reviewers completed a pilot calibration exercise to ensure consistent application of eligibility criteria. Four reviewers (YT, KM, JH, AM) took part in the screening process. Two reviewers independently assessed each citation at both stages. We resolved discrepancies through discussion and consensus.

We documented the reasons for exclusion at the full-text stage. The PRISMA-ScR flow diagram [11] presents the study selection process.

### 2.6. Data Charting and Extraction

Data were extracted using a structured charting form developed iteratively in accordance with JBI guidance. The extraction framework was informed by the parental information behaviour and illness trajectory model described by De Rouck and Leys [4,8], and by observational studies of prognostic communication in the NICU [8].

Extracted study characteristics included authorship, publication year, country, study design, population, sample size, and key findings.

Communication-related data included communication modality (verbal, written, or digital), interaction style (active, passive, individual, or collective), communication goals (information sharing or decision-making), informational content, prognostic focus, framing, and source of information. We also categorized communication based on the reported illness trajectory, which included periods of stability, gradual change, or clinical deterioration [4,15].

Prognosis in this study refers to the anticipated course or trajectory of a disease or health condition and the likelihood of future health outcomes over time, including deterioration, improvement or recovery, complications, functional outcomes, recurrence, and survival [16,17]. We synthesized communication characteristics on prognosis in the included studies using a structured framework [8]. The framework examined when prognoses were given, what outcomes clinicians discussed, how they focused and framed prognostic information, how explicit they were about prognoses, and whether they assured families of continued support.

### 2.7. Data Analysis

Data were synthesized using descriptive and thematic approaches. We organized the extracted findings according to illness trajectory, communication modality, interaction style, informational content, and parent-reported outcomes.

Thematic analysis allowed us to identify recurring patterns and gaps in descriptions of illness severity communication across studies. Analysis focused on parental engagement, communication goals, informational content, framing, and differences across the infant’s clinical course. Our review team discussion iteratively refined the themes [3].

## 3. Results

### 3.1. Study Selection and Characteristics

**Figure 1:**
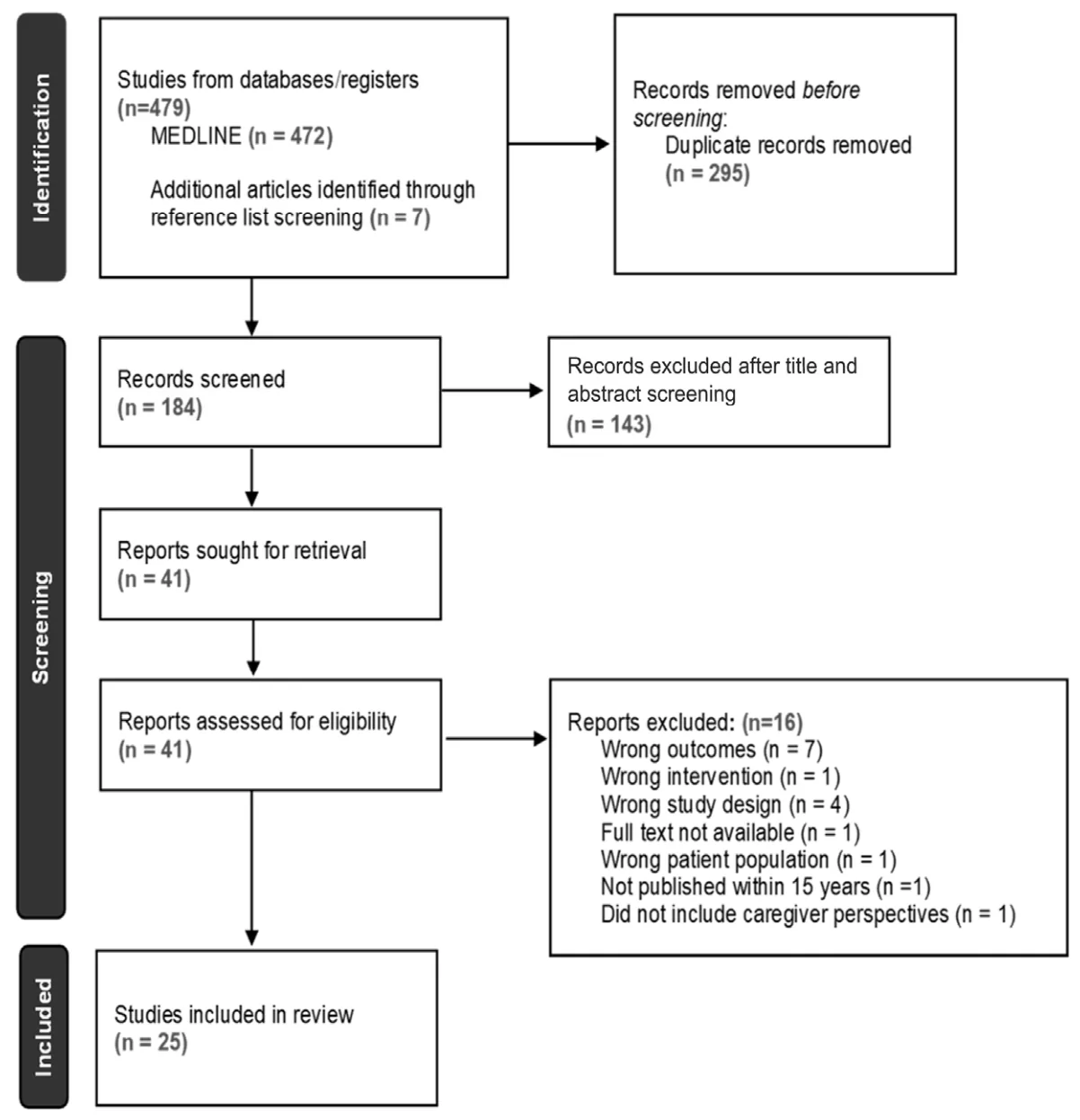
PRISMA-ScR flow diagram of study selection.

A total of 479 records were identified (472 through database searching, and 7 through reference list screening). After removing duplicates (n = 184), we excluded 143 studies after title and abstract screening because they did not meet the inclusion criteria (n = 143). We then assessed 41 full-text articles for eligibility. A total of 25 studies met inclusion criteria and were included in the final synthesis (Figure 1).

Included studies were published between 2010 and 2025 and were conducted primarily in the United States (n = 14) [8,18,19,20,21,22,23,24,25,26,27,28,29,30] alongside studies from Canada [31], Australia [32], New Zealand [33], Brazil [34], South Korea [35], Israel [36], and several European and African countries [9,10,37,38,39] (Table 1). Study designs included qualitative (n = 10) [19,24,25,26,27,32,34,37,38,39], mixed-method (n = 9) [8,9,20,21,23,28,30,31,33], and quantitative approaches (n = 6) [10,18,22,29,35,36]. Sample sizes ranged from 10 to 729 participants. Most studies involved parents of infants currently admitted to the NICU (n = 14) [9,18,20,21,22,24,29,30,33,34,35,36,37,39], a smaller number examined caregivers retrospectively following NICU discharge (n = 6) [10,23,25,31,32,38], and a few examined caregivers both during NICU admission and after discharge (n = 5) [8,19,26,27,28].

### 3.2. Communication Context and Illness Trajectory

Two articles addressed both gradually evolving trajectories and acute deterioration [10,29] while the remaining studies focused exclusively on gradually evolving conditions, with limited explicit attention to crisis situations [18,20,21,22,23,26,27,30,31,32,33,34,35,36]. Multiple studies did not report prognostic context, or it was unclear (n = 12) [18,20,21,22,24,28,29,30,32,35,36,37]. Table 2 provides references to specific studies. Although all included studies addressed aspects of illness severity communication, few studies examined how communication differed across phases of the infant’s clinical course.

### 3.3. Communication Modalities and Interaction Structures

Table 2 summarizes information on communication, including various approaches, channels, purposes, and locations, as well as interaction styles and informational content across the included studies.

Communication was predominantly clinician-led (n = 16) [8,10,20,21,22,23,24,25,26,27,28,30,33,35,37,39] and often characterized by a combination of passive and active information sharing from healthcare professionals to parents (n = 11) [8,19,24,25,28,33,34,35,37,38,39]. Eight studies reported a passive communication approach where clinicians relayed information to parents without having an interactive component [18,23,27,29,30,31,32,36] while an additional six studies described active parental engagement, involving questioning or participation [9,10,20,21,22,26].

Communication interactions were primarily collective, occurring during multidisciplinary family meetings or rounds involving multiple providers and family members (n = 11) [8,24,25,27,28,33,34,35,37,38,39], or individual, occurring between a parent and a clinician (n = 10) [9,10,18,20,21,26,29,30,31,36]. Combined interaction structures were less frequently described (n = 2) [22,23], and the two studies did not clearly specify the interaction format [19,32].

Communication primarily focused on providing updates about the infant’s condition (n = 12) [10,18,20,21,22,26,28,29,30,34,36,39], with fewer studies explicitly centred on decision-making (n = 2) [9,38]. Several studies described both information and decisional elements without clearly distinguishing between them (n = 11) [8,19,23,24,25,27,31,32,33,35,37].

Most communication occurred through face-to-face verbal interactions (n = 15) [8,9,19,22,23,24,25,28,30,33,34,35,37,38], with communication typically taking place within the NICU (n = 18) [8,9,19,20,22,24,25,27,28,29,30,31,33,34,35,36,37,38]. However, few studies described the context of these interactions. They did not specify if communication happened at the, bedside, or in a private meeting room. The studies also failed to mention whether discussions were formal, scheduled meetings or informal bedside updates. Some studies described supplementary formats such as written materials, including educational handouts and printed summaries (n = 3) [18,29,38], electronic tools (n = 4) [31,33,36,38], or audiovisual displays (n = 4) [10,18,21,22], though these approaches were inconsistently evaluated. Four studies did not report the use of a specific communication channel (n = 4) [20,26,27,32].

### 3.4. Content of Information Communicated

A total of 10 studies reported communication that integrated all three domains (biomedical, treatment-related, and care-related information) [19,21,23,24,25,26,31,32,35,39], while others described more limited content, including biomedical information alone (n = 7) [8,10,18,27,28,29,34], biomedical combined with treatment (n = 3) [20,30,32], or biomedical combined with care-related information (n = 3) [9,22,37]. Few studies focused exclusively on care-related information (n = 1) [36] or treatment and care without explicit biomedical context (n = 1) [38].

### 3.5. Decision-Making Approaches

No studies explicitly reported the use of established frameworks such as the SHARE approach (Seek patient participation, Help explore options, Assess values, Reach a decision, Evaluate the decision) or other named decision-making models [40]. Four studies described locally developed or unspecified approaches [9,19,31,38], while the majority (n = 14) did not report any structured framework guiding communication or decision-making processes [10,18,20,21,22,23,26,27,29,30,32,36,37,39].

### 3.6. Prognostic Communication in the NICU

Thirteen studies addressed prognostic communication in the NICU [8,9,10,19,23,25,26,27,31,33,34,38,39], while 12 studies either did not report or were unclear about reporting prognostic context [18,20,21,22,24,28,29,30,32,35,36,37]. Table 3 summarizes these findings, and Section 3.9 discusses them further. Seven studies described prognostic discussions addressing survival, functional outcomes (e.g., neurodevelopmental status or long-term disability), and quality of life [8,9,19,25,26,27,38]. In contrast, six studies presented more limited prognostic information, focusing on one or two of these domains [10,23,32,33,34,39].

Framing of prognostic information was most described as neutral (n = 3) [9,25,26]. Two studies reported the use of a balanced approach, using both optimistic and pessimistic framing [19,39]. Two other studies reported a pessimistic approach [23,27], and two studies presented mixed findings where participants held differing views on the framing used within the same study [8,10]. One study reported exclusively optimistic framing [34], while three studies did not specify framing [32,33,38].

### 3.7. Representation of Illness Severity

Researchers used a variety of constructs to communicate illness severity, rather than a standardized severity scale, in the included studies. The most common approach relied on clinicians’ overall assessment of the infant’s clinical status, described using qualitative terms such as stable, improving, worsening, or critically ill [24,33,37,39]. Six studies also used quantitative measures to describe illness severity [8,20,28,29,30,31]. Among these six, four used Likert scales ranging from not sick to sick (3–5 point Likert scales) [8,20,28,30]. Several studies communicated illness severity through disease-specific diagnoses or complications, including patent ductus arteriosus, cerebral palsy, brain injury, and extreme prematurity, rather than by using an overall measure of illness severity [18,24,27].

Prognostic information made up another common representation of illness severity. Studies described prognosis in terms of expected neurological outcomes based on brain imaging, neurodevelopmental impairment, survival probability, disability, and anticipated quality of life [8,9,10,23,27,39]. Illness severity was also communicated through descriptions of acute clinical deterioration requiring escalation of treatment or end-of-life discussions [9,19,25]. Less commonly, clinicians represented severity using continuous physiologic information such as bedside vital signs [29,36], explicit discussion of prognostic uncertainty [8,9,26], or the severity thresholds that prompted treatment decisions such as surgery, resuscitation, or limitation of care [31,35,38].

Overall, no study reported the use of a standardized illness severity scale specifically designed for communication with parents. Instead, clinicians used a diverse set of descriptions to represent illness severity. These included clinical status, diagnosis-specific information, physiological findings, prognostic estimates, uncertainty, and treatment decision thresholds, with the specific representation varying based on the clinical context.

### 3.8. Evidence-Derived Strategies to Improve Illness Severity Communication

We identified several evidence-derived strategies across the included studies to improve the communication of illness severity between clinicians and parents. The included studies identified specific strategies, and Supplementary Table S3 lists their references. These strategies emphasized delivering information using multiple complementary communication modalities, including written, electronic, audiovisual, and remote communication tools, to supplement rather than replace face-to-face clinician discussions. Studies also highlighted the importance of using plain language, confirming parental understanding, and actively involving parents in communication by encouraging questions and participation. Communication became more effective when clinicians explicitly acknowledged prognostic uncertainty, presented both favourable and unfavourable potential outcomes, and combined biomedical information with treatment plans and caregiving guidance. Several studies further emphasized the importance of tailoring communication to the infant’s clinical circumstances and family values, distinguishing routine information sharing from decision-making conversations, delivering serious news with empathy, maintaining consistency across members of the healthcare team, and supporting parents throughout the communication process (Supplementary Table S3).

### 3.9. Evidence-Derived Strategies to Improve Prognostic Communication

Studies examining prognostic communication identified several strategies that may improve discussions about anticipated outcomes between clinicians and parents. We provide references to specific strategies from included studies in Supplementary Table S4. Effective prognostic communication involved an individualized and iterative process that evolved throughout the infant’s clinical course, rather than a single disclosure. Recommended approaches included tailoring discussions with parents’ informational needs, balancing honesty with realistic hope, communicating the full range of potential outcomes, distinguishing population-based estimates from individualized prognosis, and explicitly discussing uncertainty. Studies also emphasized revisiting prognosis as new clinical information became available, using treatment trials with predefined goals and reassessment plans when uncertainty remained high, and clearly distinguishing prognostic discussions from shared decision-making conversations. Additional strategies included using understandable language, maintaining continuity across clinicians, responding to parents’ emotional needs, providing reassurance of continued care irrespective of prognosis, and using written or visual materials to support, but not replace, clinician–parent discussions (Supplementary Table S4).

### 3.10. Training Clinicians to Improve Parent Communication in the NICU

The included studies suggest that clinician training should focus on developing specific, observable communication skills across the continuum of illness severity. Core competencies include eliciting parents’ informational preferences and desired level of involvement [26,38,39], explaining the infant’s current clinical status and changes over time using plain language [20,30,39], communicating prognosis and uncertainty without creating false precision [8,9,26], using balanced and hope-preserving framing [8,10,19], responding appropriately to parents’ emotional needs [8,25,34], distinguishing information sharing from decision support [9,31,38], and establishing explicit plans for reassessment when prognosis remains uncertain [9,19]. Collectively, these findings support the development of educational programmes that emphasize experiential learning and opportunities for feedback to strengthen these communication competencies, while promoting the appropriate use of written, visual, and digital communication tools to complement, rather than replace, direct clinician–parent interactions [18,21,22,29,36].

### 3.11. Gaps in Reporting Across Studies

Studies inconsistently described several aspects of illness severity communication, such as parental participation, prognostic framing, illness trajectory, and communication goals. Most studies focused on clinician-led verbal communication, with limited attention to digital communication tools, parent-centred outcomes, or contextual factors that may influence communication experiences.

## 4. Discussion

### 4.1. Overall Interpretation

This review found that illness severity communication in the NICU is predominantly clinician-led, biomedical in focus, and centred on information delivery rather than interactive discussion or shared decision-making. Researchers seldom analyzed communication across phases of the infant’s clinical course, and few studies clearly differentiated between communication intended to provide clinical updates and communication intended to support decision-making. The studies also inconsistently described prognostic framing, parental engagement, and communication modalities. Together, these findings suggest that, although NICU communication is a widely studied topic, inconsistent definitions and poor integration across clinical contexts prevent effective approaches to illness severity communication.

### 4.2. Parental Information Behaviour

Parents were most often described as recipients of information rather than active participants in communication. Although some studies reported parental questioning or participation in discussions, few examined how parents seek, interpret, or use information throughout their stay in the NICU while their infant experiences changing severity of illness.

The framework by De Rouck and Leys may help address this gap by situating parental information needs within the infant’s illness trajectory [4]. This approach recognizes that informational needs and engagement may change across periods of stability, deterioration, and recovery, and may support more responsive communication practices in the NICU.

### 4.3. Information Sharing vs. Decision Support

Many studies described communication that combined information sharing and decision-making without clearly distinguishing between the two. This lack of clarity may obscure important differences between communication intended to provide clinical understanding and communication intended to support shared decision-making.

Observational work by Boss et al. highlights communication practices that may support prognostic discussions, including clarity, balanced framing, and responsiveness to parental cues [1]. Greater distinction between informational and decisional communication may help align communication strategies with parental needs and clinical context.

### 4.4. Temporal Context and Illness Trajectory

Few studies examined how communication changed across the infant’s clinical trajectory, such as during stabilization, deterioration, recovery, or end-of-life care. Researchers often presented communication of illness severity as static, and not responsive to changes in illness severity or prognosis.

Although De Rouck and Leys’ [4] and Boss et al.’s [1] conceptual frameworks implicitly highlight the significance of context and timing, the literature has not systematically applied them. An illness trajectory perspective may help situate communication within evolving clinical contexts and better reflect changes in parental informational and emotional needs over time.

### 4.5. Content and Framing

Communication about illness severity focused primarily on biomedical information, while treatment-related and care-related discussions were less consistently included. Prognostic communication also varied substantially across studies. Researchers commonly discussed survival, but they addressed long-term functional outcomes and quality of life less consistently.

Communication of uncertainty was often inconsistent, and unclear timeframes frequently marked prognostic discussions. These inconsistencies may affect how parents interpret the prognosis and understand the infant’s clinical course.

Despite evidence that framing may influence parental understanding, expectations, and decision-making, researchers rarely examined prognostic information framing in depth [8,10]. Few studies have evaluated how different communication approaches affect parent-centred outcomes such as comprehension, emotional impact, or decision-making.

### 4.6. Toward an Integrated Framework

Existing literature lacks a consistent framework for illness severity communication in the NICU. De Rouck and Leys [4] emphasize parental information behaviour across the illness trajectory, while Boss et al. [1] describe communication practices relevant to prognostic discussions. Several other studies offer insight into parental preferences in prognosis communication [8,19,23,41]. Integrating these approaches may support a more structured understanding of illness severity communication across changing clinical contexts.

A trajectory-based model may also help align communication strategies with periods of stabilization, deterioration, recovery, or end-of-life care, recognizing that parental informational and emotional needs may change over time.

### 4.7. Comparison with Existing Reviews

Previous reviews of NICU communication have focused primarily on parental experiences, emotional support, and shared decision-making [42,43]. While these studies emphasize the importance of family-centred communication, they rarely examine how communication differs across illness trajectories or distinguish between structural aspects of communication such as framing, informational content, and communication goals.

In contrast, this review synthesizes illness severity communication across multiple dimensions, including parental engagement, framing, informational content, and illness trajectory.

### 4.8. Strengths

A major strength of this review is that it moves beyond describing individual communication interventions to examine the conceptual structure of illness severity communication in the NICU. Previous studies have largely focused on evaluating communication modalities, such as verbal discussions, telemedicine, text messaging, audiovisual education, electronic medical record summaries, and decision-support tools. In contrast, this review synthesizes the content of illness severity communication itself and demonstrates that there is no consistent framework for organizing information communicated to parents.

This review provides a structured synthesis of illness severity communication in the NICU across domains, including parental engagement, informational content, framing, and illness trajectory. Using conceptual frameworks helped identify recurring patterns and gaps across studies. Including qualitative, quantitative, and mixed-method studies also allowed for a broad overview of communication practices.

### 4.9. Limitations

There are several limitations. Heterogeneity across studies limited direct comparison of findings, and many studies lacked detailed reporting on communication timing, framing, and modality. Most studies took place in high-income settings, potentially limiting generalizability. In addition, we limited the search primarily to MEDLINE and English-language studies, which may have excluded relevant literature indexed elsewhere and limited the validity and credibility of the review findings. We did not perform an updated literature search prior to manuscript submission.

### 4.10. Implications

These findings highlight the need for clearer approaches to illness severity communication in the NICU, particularly across different phases of the infant’s clinical course. Greater attention to parental engagement, prognostic framing, and distinctions between informational and decisional communication may help improve communication practices and better support families during NICU care. By integrating findings across diverse study designs, this review proposes a structured conceptual model that distinguishes four complementary domains of illness severity communication: current clinical status, change from prior status (clinical trajectory), forecasting of anticipated clinical course and prognosis, and decision support when treatment choices arise. This framework, unlike existing approaches that primarily address how information is delivered, focuses on what information to communicate and when to communicate it across the infant’s clinical course. This distinction addresses an important conceptual gap in the literature and provides a foundation for future research, communication tool development, and the evaluation of parent-centred communication strategies.

Although the included studies identify several promising strategies, we should approach their widespread adoption cautiously. Most evidence came from small, single-centre, observational, qualitative, preference, or feasibility studies, which limited their assessment of comprehension, recall, emotional impact, decision quality, equity, or clinical outcomes. Digital, written, and structured communication approaches should therefore complement rather than replace relational clinician–parent communication and should undergo further evaluation across diverse populations, clinical phases, and levels of illness severity before routine implementation.

## 5. Conclusions

Clinicians predominantly lead NICU illness severity communication, which is heterogeneous and lacks a consistent framework for guiding information dissemination as an infant’s condition changes. This review synthesizes the existing literature into key domains of communication, including parental engagement, communicative purpose, informational content, prognostic framing, and illness trajectory, providing a structured foundation for future research and practice. Greater standardization of illness severity communication, together with approaches that better support parental understanding, engagement, and shared decision-making, may improve family-centred care in the NICU.

## Figures and Tables

**Table 1:** Characteristics of included studies examining illness severity communication in the neonatal intensive care unit (NICU).

Author (Year of Publication)	Country of Study	Study Design *	Study Details	Population, Timing	Sample Size ^@^ [Parents (% Female); Clinicians]
Alur et al. (2010) [18]	United States	Quantitative	Survey	Parents during NICU admission	Parents: n = 46 (60.9% female)
Arnolds et al. (2018) [19]	United States	Qualitative	Interviews	Parents during NICU admission and after discharge or death	During admission: 26 families (37 parents); follow-up: 17 mothers
Boadu et al. (2024) [37]	Ghana	Qualitative	Interviews	Parents during NICU admission	Parents: n = 16 (81%)
Boss et al. (2010) [30]	United States	Mixed methods	Survey and interviews	Adolescent mothers during NICU admission	Parents: n = 42 (100%)
Boss et al. (2017) [8]	United States	Mixed methods	Recorded conversations and surveys	Parents and clinicians during NICU admission; parents also surveyed after discharge	Parents: n = 31 (61%); clinicians: n = 23
Choi et al. (2025) [35]	South Korea	Quantitative	Survey	Parents during NICU admission	Parents: n = 103 (84%)
De Proost et al. (2025) [38]	Netherlands	Qualitative	Interviews	Parents after NICU discharge	Parents: n = 19 (63%)
de Wit et al. (2013) [20]	United States	Mixed methods	Interviews	Mothers and clinicians during NICU admission	Parents: n = 101 (100%); clinicians: n = 101
Epstein et al. (2015) [21]	United States	Mixed methods	Survey and free-text analysis	Parents and clinicians during NICU admission	Parents: n = 26 (86%); clinicians: n = 37
Forth et al. (2024) [10]	Germany	Quantitative	Randomized crossover trial using video vignettes and surveys	Parents after NICU discharge	Parents: n = 220 (64.5%)
Fowler et al. (2019) [32]	Australia	Qualitative	Interviews	Mothers after NICU discharge	Parents: n = 10 (100%)
Frize et al. (2013) [31]	Canada	Mixed methods	Survey and free-text analysis	Parents after NICU discharge and neonatologists	Parents: n = 8 (NR); clinicians: n = 5
Garingo et al. (2016) [22]	United States	Quantitative	Survey	Parents and clinicians during NICU admission	Parents: n = 9 (NR); clinicians: n = 10; infants: n = 40
Globus et al. (2016) [36]	Israel	Quantitative	Survey	Parents and nurses during NICU admission	Parents: pre-intervention n = 91; post-intervention n = 87; nurses: pre-intervention n = 27; post-intervention n = 35
Guttmann et al., (2020) [23]	United States	Mixed methods	Survey and free-text analysis	Parents of children with cerebral palsy after NICU discharge	Parents: n = 463 (NR); free-text respondents: n = 266
Guttmann et al. (2024) [24]	United States	Qualitative	Interviews	Parents during NICU admission	Parents: n = 14 (NR)
Harvey et al. (2013) [39]	United Kingdom	Qualitative	Interviews	Parents during NICU admission	Parents: n = 18 (72%)
Krick et al. (2020) [26]	United States	Qualitative	Interviews	Parents during NICU admission and after discharge	Parents: n = 24 (75%)
Kukora et al. (2025) [25]	United States	Qualitative	Interviews	Parents after NICU discharge	Parents: n = 15 (NR)
Lemmon et al. (2019) [27]	United States	Qualitative	Interviews	Parents during NICU admission and after discharge; clinicians	Parents: n = 16 (NR); clinicians: n = 53
Limacher et al. (2023) [9]	Switzerland	Mixed methods	Recorded conversations and medical record review	Parents and clinicians during NICU admission	Parents: n = 16 (50%); clinicians: NR; families: n = 8; conversations: n = 16
Malin et al. (2020) [28]	United States	Mixed methods	Interviews and medical record review	Parents and clinicians during NICU admission and after discharge	Parents: n = 162 (88%); clinicians: n = 325
Marçola et al. (2020) [34]	Brazil	Qualitative	Interviews	Parents of infants with congenital malformations during NICU admission	Parents: n = 30 (93%)
Palma et al. 2012 [29]	United States	Quantitative	Survey	Parents during NICU admission	Families: pre-intervention n = 31; post-intervention n = 26
Williams et al. (2020) [33]	New Zealand	Mixed methods	Survey and free-text analysis	Parents during NICU admission	Parents: n = 41 (62%)

* Study designs were categorized as qualitative, quantitative, or mixed-method; NR, not reported. Percentages indicate the proportion of mothers unless the study. ^@^ Parent, family, clinician, infant, and conversation samples are identified separately where applicable.

**Table 2:** Communication characteristics of studies examining illness severity communication in the neonatal intensive care unit (NICU).

Author (Year)	Communication Approach ^1^	Interaction Type ^2^	Communication Channel	Communication Purpose	Information Content	Communication Location	Source of Communication	Prognosis Communicated	Illness Severity Measure
Alur et al. (2010) [18]	Passive	Individual	Written; Audiovisual	Information sharing only	Biomedical	NA	NA	No	NR
Arnolds et al. (2018) [19]	Combined	NR	Oral	Both	Biomedical. Treatment. Care	NICU	NR	Yes	NR
Boadu et al. (2024) [37]	Combined	Collective	Oral	Both	Biomedical. Care	NICU	Clinicians ^3^	No	NR
Boss et al. (2010) [30]	Passive	Individual	Oral	Information sharing only	Biomedical. Treatment	NICU	Clinicians ^4^	NR	Yes (3-point Likert scale) ^7^
Boss et al. (2017) [8]	Combined	Collective	Oral	Both	Biomedical	NICU	Clinicians	Yes	Yes ^8^ (estimated likelihood)
Choi et al. (2025) [35]	Combined	Collective	Oral	Both	Biomedical. Treatment. Care	NICU	Clinicians ^4^	NR	NR
De Proost et al. (2025) [38]	Combined	Collective	Oral; Written; Electronic	Decision-making	Treatment. Care	Both	NR	Yes	NR
de Wit et al. (2013) [20]	Active	Individual	NR	Information sharing only	Biomedical. Treatment	Both	Clinicians	NR	Yes (4-point scale) ^7^
Epstein et al. (2015) [21]	Active	Individual	Audiovisual	Information sharing only	Biomedical. Treatment. Care	Virtual	Clinicians	NR	NR
Forth et al. (2024) [10]	Active	Individual	Audiovisual	Information sharing only	Biomedical	NR	Clinicians ^5^	Yes	NR
Fowler et al. (2019) [32]	Passive	NR	NR	Both	Biomedical. Treatment	Virtual	NR	NR	NR
Frize et al. (2013) [31]	Passive	Individual	Electronic	Both	Biomedical. Treatment. Care	Both	NR	Yes	Yes (Speedometer method) ^9^
Garingo et al. (2016) [22]	Active	Both	Oral; Audiovisual	Information sharing only	Biomedical. Care	NICU ^6^	Clinicians	NR	NR
Globus et al. (2016) [36]	Passive	Individual	Electronic	Information sharing only	Care	Both	NA	NR	NR
Guttmann et al. (2020) [23]	Passive	Both	Oral	Both	Biomedical. Treatment. Care	NR	Clinicians	Yes	NR
Guttmann et al. (2024) [24]	Combined	Collective	Oral	Both	Biomedical. Treatment. Care	NICU	Clinicians	NR	NR
Harvey et al. (2013) [39]	Combined	Collective	Oral	Information sharing only	Biomedical. Treatment. Care	NR	Clinicians ^4^	Yes	NR
Krick et al. (2020) [26]	Active	Individual	NR	Information sharing only	Biomedical. Treatment. Care	NR	Clinicians ^4^	Yes	NR
Kukora et al. (2025) [25]	Combined	Collective	Oral	Both	Biomedical. Treatment. Care	NICU	Clinicians	Yes	NR
Lemmon et al. (2019) [27]	Passive	Collective	NR	Both	Biomedical	NICU	Clinicians	Yes	NR
Limacher et al. (2023) [9]	Active	Individual	Oral	Decision-making	Biomedical. Care	NICU	NR	Yes	NR
Malin et al. (2020) [28]	Combined	Collective	Oral	Information sharing only	Biomedical	NICU	Clinicians ^4^	NR	Yes (5-point Likert scale) ^7^
Marçola et al. (2020) [34]	Combined	Collective	Oral	Information sharing only	Biomedical	NICU	NR	Yes	NR
Palma et al. (2012) [29]	Passive	Individual	Written	Information sharing only	Biomedical	NICU	NR	NR	Yes (descriptive such as well/unwell)
Williams et al. (2020) [33]	Combined	Collective	Oral. Written. Electronic	Both	Biomedical. Treatment. Care	NICU	Clinicians ^4^	Yes	NR

^1^ = Communication approach: Active communication means parents helped guide the discussion by asking questions or requesting information. Passive communication means clinicians or communication systems provided information with limited input from parents. Combined communication included both active and passive communication; ^2^ = Interaction type: Individual communication involved one healthcare professional. Collective communication involved a healthcare team. Both included individual and team communication; ^3^ = Clinicians refer to one or more health care providers such as doctors, nurses, social worker, managers, respiratory therapists, physician assistants, trainees, or nurse practitioners; ^4^ = Clinicians were specifically described as physicians and nurses in the study; ^5^ = Clinicians were specifically described as physicians in the study; ^6^ = Communication occurred in the NICU with an on-site clinical team and an attending neonatologist participating remotely through robotic tele-rounding; ^7^ = Illness severity was measured using a Likert-type scale ranging from “not sick” to “very sick,” or a comparable scale; ^8^ = Prognosis was reported as the estimated likelihood of survival without serious health problems: 0–10%, 11–25%, 26–50%, 51–75%, or >75%.; ^9^ = Risk of mortality and other adverse outcomes were displayed using a graphical “speedometer” ranging from 0% to 100%: low risk, 0–30%; moderate risk, 31–70%; and high risk, 71–100%. The predicted risk was also presented in sentence form (e.g., “For 100 babies with problems similar to your baby’s, 85 will die” for a predicted risk of 85%). NA = not applicable, NR = not reported.

**Table 3:** Communication characteristics of studies examining prognostic communication in the NICU.

Study	Prognosis Timeframe ^1^	Trajectory and Reassessment ^2^	Prognosis Topic ^3^	Prognosis Focus ^4^	Affective Framing ^5^	Framing Orientation ^6^	Explicitness of Prognosis ^7^	Assurance of Continued Care ^8^
Arnolds et al. (2018) [19]	Short-; Mid-; Long-term	Trials of treatment and reassessment over time were central to parents’ experiences	Survival; Functional outcomes; Quality of life	Individualized	Balanced	Uncertainty-centred; Family-centred; Capability-centred; Risk-centred	Broad	Present
Boss et al. (2017) [8]	Short-; Mid-; Long-term	Immediate risks and anticipated clinical course were discussed variably; reassessment and treatment implications were sometimes addressed	Survival; Functional outcomes; Quality of life	Both	Mixed	Medicalized; Risk-centred; Impairment-centred; Uncertainty-centred; Family-centred	Both	Present
De Proost et al. (2025) [38]	Short-; Long-term	Treatment options at birth and response to initial intensive care were considered; parents supported reassessment and personalized decisions	Survival; Functional outcomes; Quality of life	Both	NR	Risk-centred; Capability-centred; Family-centred; Uncertainty-centred; Medicalized	Both	NR
Forth et al. (2024) [10]	Long-term	NR	Survival; Functional outcomes	Population-based	Mixed	Risk-centred; Impairment-centred; Capability-centred; Medicalized	Precise	NR
Frize et al. (2013) [31]	Short-; Mid-; Long-term	Current condition, current treatment, trends, alerts, risk prediction and decision support	Survival; Functional outcomes	Individualized	NR	Risk-centred; Medicalized	Precise	NR
Guttmann et al. (2019) [23]	Long-term	NR	Functional outcomes; Quality of life	Individualized	Pessimistic	Impairment-centred; Medicalized; Risk-centred	Both	NR
Harvey et al. (2013) [39]	Long-term	NR	Functional outcomes; Quality of life	Both	Balanced	Impairment-centred; Uncertainty-centred; Family-centred; Medicalized	Broad	Present
Krick et al. (2020) [26]	Short-; Mid-; Long-term	Parents described monitoring the infant’s course and adjusting expectations as new information emerged	Survival; Functional outcomes; Quality of life	Individualized	Neutral	Uncertainty-centred; Family-centred; Capability-centred	Broad	Present
Kukora et al. (2025) [25]	Short-; Long-term	Serious news influenced subsequent care and decision-making; parents valued explanation of what would happen next	Survival; Functional outcomes; Quality of life	Individualized	Neutral	Family-centred; Uncertainty-centred; Risk-centred; Medicalized	Both	Present
Lemmon et al. (2019) [27]	Long-term	NR	Survival; Functional outcomes; Quality of life	Both	Pessimistic	Impairment-centred; Capability-centred; Family-centred; Medicalized	Broad	NR
Limacher et al. (2023) [9]	Short-; Long-term	Possible improvement, deterioration or death; treatment continuation; repeated reassessment; options were often delayed until deterioration or poor prognosis became clearer	Survival; Functional outcomes; Quality of life	Both	Neutral	Uncertainty-centred; Risk-centred; Impairment-centred; Medicalized; Family-centred	Broad	Present
Marçola et al. (2020) [34]	Short-; Long-term	NR	Survival; Functional outcomes	Individualized	Optimistic	Family-centred; Medicalized; Risk-centred	Broad	Present
Williams et al. (2020) [33]	NR	NR	Functional outcomes	Individualized	NR	Family-centred; Medicalized	Broad	NR

^1^ = Prognosis timeframe: Short-term refers to the next week, mid-term refers to the period before NICU discharge, and long-term refers to the period after discharge.; ^2^ = Trajectory and reassessment: Describes anticipated clinical changes, treatment implications, and plans to reassess prognosis.; ^3^ = Prognosis topic: Survival, functional outcomes, or quality of life.; ^4^ = Prognosis focus: Population-based, individualized, or both.; ^5^ = Affective framing: Optimistic emphasizes favourable outcomes; pessimistic emphasizes unfavourable outcomes; balanced presents both favourable and unfavourable outcomes; neutral emphasizes neither; and mixed indicates that different affective framing were reported by participants in a single study. Experimental framing indicates framing presented as part of a simulated study; ^6^ = Framing orientation: Impairment-centred emphasizes disability or functional limitations; risk-centred emphasizes probabilities or adverse outcomes; uncertainty-centred emphasizes limits of prediction; capability-centred emphasizes survival, abilities, or favourable outcomes; family-centred includes family values, experiences, or impacts; and medicalized emphasizes diagnoses, tests, treatments, or clinical needs; ^7^ = Explicitness of prognosis: Broad refers to general or qualitative descriptions; precise refers to numerical or model-based estimates; and both indicates that broad and precise information was reported; ^8^ = Assurance of continued care: Present indicates that clinicians communicated ongoing care, support, or partnership; absent indicates that this assurance was not provided; NR, not reported.

## Data Availability

No new data were created or analyzed in this study. Data sharing is not applicable to this article.

## Supplementary Materials

The following supporting information can be downloaded at https://www.mdpi.com/article/10.3390/children13081006/s1, Supplementary Table S1: Preferred Reporting Items for Systematic reviews and Meta-Analyses extension for Scoping Reviews (PRISMA-ScR) Checklist [44,45]. Supplementary Table S2: MEDLINE (Ovid) search strategy. Supplementary Table S3: Evidence-derived strategies for improving illness-severity communication in the NICU. Supplementary Table S4: Evidence-derived strategies for improving prognosis communication with parents in the NICU.

## Abbreviations

The following abbreviations are used in this manuscript:

NICU	Neonatal Intensive Care Unit
JBI	Joanna Briggs Institute
PRISMA-ScR	Preferred Reporting Items for Systematic Reviews and Meta-Analyses Extension for Scoping Reviews
PICO	Population, Intervention, Comparison, Outcome
Ovid	Ovid database platform
SMS	Short Message Services
SHARE	Seek patient participation, Help explore options, Assess values, Reach a decision, Evaluate the decision
MEDLINE	Medical Literature Analysis and Retrieval System Online
