# Supplementary material for: Communication of Illness Severity to Parents in the Neonatal Intensive Care Unit: A Scoping Review of Communication Characteristics and Prognostic Framing
Source: Children (Basel). 2026 Jul 29;13(8):1006. doi: 10.3390/children13081006 (PMC13511270; doi:10.3390/children13081006)
Supplement: Supplementary file 1 [file children-13-01006-s001.zip › children-4364629-supplementary.pdf]

Supplementary Table S1: Preferred Reporting Items for Systematic reviews and Meta-Analyses extension for Scoping Reviews (PRISMA-ScR) Checklist

SECTION	ITEM	PRISMA-ScR CHECKLIST ITEM	REPORTED ON PAGE #
		**TITLE**	
Title	1	Identify the report as a scoping review.	1
		**ABSTRACT**	
Structured summary	2	Provide a structured summary that includes (as applicable): background, objectives, eligibility criteria, sources of evidence, charting methods, results, and conclusions that relate to the review questions and objectives.	1-2
		**INTRODUCTION**	
Rationale	3	Describe the rationale for the review in the context of what is already known. Explain why the review questions/objectives lend themselves to a scoping review approach.	2
Objectives	4	Provide an explicit statement of the questions and objectives being addressed with reference to their key elements (e.g., population or participants, concepts, and context) or other relevant key elements used to conceptualize the review questions and/or objectives.	2
		**METHODS**	
Protocol and registration	5	Indicate whether a review protocol exists; state if and where it can be accessed (e.g., a Web address); and if available, provide registration information, including the registration number.	3
Eligibility criteria	6	Specify characteristics of the sources of evidence used as eligibility criteria (e.g., years considered, language, and publication status), and provide a rationale.	4
Information sources*	7	Describe all information sources in the search (e.g., databases with dates of coverage and contact with authors to identify additional sources), as well as the date the most recent search was executed.	3
Search	8	Present the full electronic search strategy for at least 1 database, including any limits used, such that it could be repeated.	3-4
Selection of sources of evidence†	9	State the process for selecting sources of evidence (i.e., screening and eligibility) included in the scoping review.	4
Data charting process‡	10	Describe the methods of charting data from the included sources of evidence (e.g., calibrated forms or forms that have been tested by the team before their use, and whether data charting was done independently or in duplicate) and any processes for obtaining and confirming data from investigators.	4

Data items	11	List and define all variables for which data were sought and any assumptions and simplifications made.	4
Critical appraisal of individual sources of evidence§	12	If done, provide a rationale for conducting a critical appraisal of included sources of evidence; describe the methods used and how this information was used in any data synthesis (if appropriate).	NA
Synthesis of results	13	Describe the methods of handling and summarizing the data that were charted.	4-5
**RESULTS**			
Selection of sources of evidence	14	Give numbers of sources of evidence screened, assessed for eligibility, and included in the review, with reasons for exclusions at each stage, ideally using a flow diagram.	5
Characteristics of sources of evidence	15	For each source of evidence, present characteristics for which data were charted and provide the citations.	5-7
Critical appraisal within sources of evidence	16	If done, present data on critical appraisal of included sources of evidence (see item 12).	NA
Results of individual sources of evidence	17	For each included source of evidence, present the relevant data that were charted that relate to the review questions and objectives.	5-7
Synthesis of results	18	Summarize and/or present the charting results as they relate to the review questions and objectives.	5-7
**DISCUSSION**			
Summary of evidence	19	Summarize the main results (including an overview of concepts, themes, and types of evidence available), link to the review questions and objectives, and consider the relevance to key groups.	9-10
Limitations	20	Discuss the limitations of the scoping review process.	11
Conclusions	21	Provide a general interpretation of the results with respect to the review questions and objectives, as well as potential implications and/or next steps.	11
**FUNDING**			
Funding	22	Describe sources of funding for the included sources of evidence, as well as sources of funding for the scoping review. Describe the role of the funders of the scoping review.	11

JBI = Joanna Briggs Institute; PRISMA-ScR = Preferred Reporting Items for Systematic reviews and Meta-Analyses extension for Scoping Reviews.

* Where *sources of evidence* (see second footnote) are compiled from, such as bibliographic databases, social media platforms, and Web sites.

† A more inclusive/heterogeneous term used to account for the different types of evidence or data sources (e.g., quantitative and/or qualitative research, expert opinion, and policy

documents) that may be eligible in a scoping review as opposed to only studies. This is not to be confused with *information sources* (see first footnote).
‡ The frameworks by Arksey and O'Malley [44] and Levac and colleagues [45] and the JBI guidance [11,12] refer to the process of data extraction in a scoping review as data charting.
§ The process of systematically examining research evidence to assess its validity, results, and relevance before using it to inform a decision. This term is used for items 12 and 19 instead of "risk of bias" (which is more applicable to systematic reviews of interventions) to include and acknowledge the various sources of evidence that may be used in a scoping review (e.g., quantitative and/or qualitative research, expert opinion, and policy document).

From: Tricco AC, Lillie E, Zarin W, O'Brien KK, Colquhoun H, Levac D, et al. PRISMA Extension for Scoping Reviews (PRISMAScR): Checklist and Explanation. Ann Intern Med. 2018;169:467–473. doi: 10.7326/M18-0850. [11]

Supplementary Table S2: MEDLINE (Ovid) search strategy.

Line	Search Strategy
1	((parent* or caregiver* or mother* or father*) adj3 (NICU or "neonatal intensive care" or "neonatal unit" or "newborn care" or "neonatal care")).mp. OR (exp Parents/ AND (exp Infant, Premature/ OR exp Infant, Low Birth Weight/ OR exp Intensive Care Units, Neonatal/))
2	("illness severity" or "disease severity" or "health status" or "clinical deterioration" or "critical illness" or "condition severity" or "severity of illness" or "disease progression" or "health condition severity" or "critical condition" or "complication severity" or "serious illness" or "advanced illness" or "health instability").mp.
3	exp Severity of Illness Index/ OR exp Health Status Indicators/ OR exp Prognosis/ OR exp Critical Illness/ OR exp Disease Progression/ OR exp Risk Assessment/
4	(communication or "information sharing" or "health communication" or "information exchange" or "knowledge sharing" or "patient-provider communication" or "provider-family communication" or "clinical communication" or "medical communication" or "interpersonal communication" or "health information sharing" or "health information exchange" or "communication strategies" or "communication practices" or "communication in healthcare" or "care communication" or "education and communication" or discussion or consultation or "health information transfer" or "written communication" or "verbal communication" or "non-verbal communication" or emoji* or chart* or graph*).mp.
5	exp Communication/ OR exp Physician-Patient Relations/ OR exp Consumer Health Information/ OR exp Patient Education as Topic/ OR exp Plain Language Summaries/
6	4 OR 5
9	2 OR 3
10	1 AND 6 AND 9

Supplementary Table S3: Evidence-derived strategies for improving illness-severity communication in the NICU

Suggested strategy	Evidence from included studies	Relevant studies
Provide regular written or electronic clinical updates to supplement	Daily electronic update letters and text-based updates were feasible and supported parental access to current information, particularly when parents	Palma et al. (2012)[29]; Globus et al. (2016)[36];

verbal communication	could not be continuously present at the bedside. These approaches should supplement rather than replace clinician discussion.	Williams (2020)[33]
Offer remote participation in clinical updates and rounds	Video communication and tele-rounding allowed parents who were physically absent to interact with clinicians and receive updates. These approaches were generally feasible and acceptable.	Epstein et al. (2015)[21]; Garingo et al. (2016)[22]
Use audiovisual explanations for complex physiologic or disease processes	Parents preferred a brief audiovisual animation over printed material for understanding patent ductus arteriosus. In the Alur study, 50% preferred animation compared with 17.4% who preferred print, and 85% reported understanding after reviewing both formats.	Alur et al. (2010)[18]
Use plain language and verify parental understanding	Studies identified gaps between what clinicians communicated and what parents understood, particularly among younger parents and during complex prognostic discussions. Communication should include opportunities for clarification and confirmation of understanding rather than assuming comprehension.	Boss et al. (2010)[30]; de Wit et al. (2013)[20]; Harvey et al. (2013)[29]
Clearly acknowledge prognostic uncertainty	Parents experienced uncertainty as a major component of the NICU course. Real-time observations showed that probabilities and uncertainty were not always communicated consistently or explicitly. Clinicians should distinguish what is known, what remains uncertain, and what may become clearer over time.	Limacher et al. (2023)[9]; Krick et al. (2020)[26]; Arnolds et al. (2018)[19]
Present both favourable and unfavourable possible outcomes	Framing influenced parental interpretation of prognosis. Parents may value communication that acknowledges serious risks while preserving the possibility of a favourable outcome, rather than communication dominated by either optimism or pessimism alone.	Forth et al. (2024)[10]; Boss et al. (2017)[8]
Discuss prognosis across several outcome domains	Parents and clinicians did not always assign the same importance to survival, disability, medical complexity, and quality of life. Prognostic communication should therefore extend beyond survival and neurological impairment alone.	Lemmon et al. (2019)[27; Guttmann et al. (2019)[23]; Harvey et al. (2013)[39]
Explore what outcomes matter most to the family	Parents' values, expectations, and definitions of an acceptable outcome varied. Communication was more useful when clinicians explored family priorities rather than relying only on clinician-selected outcomes.	Lemmon et al. (2019)[27]; De Proost et al. (2025)[38]; Arnolds et al. (2018)[19]
Personalize communication rather than relying only on	Parents valued individualized discussion that considered their infant's circumstances and their family's values. Population-level estimates or	De Proost et al. (2025)[38]; Forth et al. (2024)[10];

generalized risk estimates or guidelines	standardized periviability information were not sufficient on their own.	Guttmann et al. (2019)[23]
Distinguish information updates from conversations requiring a decision	Several studies demonstrated that parents may receive prognostic or clinical information without a clearly identified decision, while other discussions require explicit deliberation about options. Clinicians should clarify whether the immediate purpose is awareness, preparation, or decision-making.	Frize et al. (2013)[31]; Limacher et al. (2023)[9]; Choi et al. (2025)[35]
Explicitly identify the decision, available options, and parental role	Parents' experiences of shared decision-making were influenced by illness uncertainty, self-efficacy, and the quality of clinician communication. Decision-focused conversations should make the decision and the parent's desired role explicit.	Choi et al. (2025)[35]; Frize et al. (2013)[31]; Kukora et al. (2025)[25]
Deliver serious news with empathy, preparation, and sufficient opportunity for questions	The way serious news was communicated affected parental experience, understanding, and subsequent decision-making. Parents valued respectful delivery, emotional support, and time to process information.	Kukora et al. (2025)[25]; Marçola et al. (2020)[34]; Fowler et al. (2019)[32]
Provide consistent messages across members of the clinical team	Parent–clinician disagreement and differing interpretations of prognosis contributed to confusion. Communication should be coordinated across clinicians while still acknowledging legitimate uncertainty or differing professional views.	de Wit et al. (2013)[20]; Boss et al. (2017)[8]; Guttmann et al. (2024)[24]
Support active parental participation and questioning	Parents should not be positioned solely as passive recipients. Studies describing stronger communication quality emphasized opportunities for parents to ask questions, contribute knowledge about their infant, and participate in discussions.	Williams (2020)33]; Guttmann et al. (2024)[24]; Boadu et al. (2024)[37]
Combine biomedical information with treatment and caregiving guidance	Parents required more than diagnostic facts. They also valued explanations of treatments, daily caregiving, comfort, and how they could participate in their infant's care.	Boadu et al. (2024)[37]; Fowler et al. (2019)[32]; Williams (2020)[33]

Footnote: Evidence-derived strategies were based on findings directly reported by included studies, including observed communication practices, parent-reported experiences or preferences, feasibility findings, and comparative intervention results. These findings should not all be interpreted as evidence of effectiveness because the included studies used heterogeneous designs.

Supplementary Table S4: Evidence-derived strategies for improving prognosis communication with parents in the NICU

Strategy	Key findings from included studies	Supporting studies
Tailor prognostic communication to parents' information needs and preferred level of detail	Parents differ in how much prognostic information they want, when they want it, and how directly they want it presented. Communication should therefore begin by asking what parents understand, what they want to know, and how they prefer information to be discussed rather than assuming that one approach suits all families.	De Proost et al. (2025)[38]; Krick et al. (2020)[26]; Harvey et al. (2013)[39]
Present prognosis honestly while preserving realistic hope	Parents value clinicians who acknowledge the possibility of poor outcomes without presenting those outcomes as inevitable. In the COPE trial, parents generally preferred optimistic framing of the same prognostic information, and optimistic framing produced more realistic expectations of survival, although not of impairment. These findings support balanced, hope-sensitive communication rather than uniformly pessimistic counselling.	Forth et al. (2024)[10]; Arnolds et al. (2018)[26]; Boss et al. (2017)[39]
Describe both favourable and unfavourable possible outcomes	Prognosis should not be framed solely around mortality or disability. Parents benefit from hearing the range of plausible outcomes, including survival, possible impairment, preserved capabilities, medical needs, and the lived experience of the child and family.	Forth et al. (2024)[10]; Lemmon et al. (2019)[27]; Guttmann et al. (2019)[23]
Discuss outcomes that matter to parents, not only clinician-selected impairments	Parents and clinicians may interpret prognosis differently. Parents in Lemmon et al. focused more on survival, hope, spirituality, and the broader future of the child, whereas clinicians emphasized neurological impairment and probabilistic risk. Clinicians should explicitly explore which outcomes parents consider most important and avoid assuming that disability necessarily represents an unacceptable quality of life.	Lemmon et al. (2019)[27]; Guttmann et al. (2019)[23]; Harvey et al. (2013)[39]
Differentiate population estimates from the individual infant's prognosis	Population-based data provide useful context but cannot precisely predict an individual infant's outcome. Clinicians should explain how published estimates apply to the infant, identify infant-specific factors that may alter prognosis, and avoid creating false precision.	Forth et al. (2024)[10]; De Proost et al. (2025)[38]; Frize et al. (2013)[31]
State uncertainty explicitly and explain its source	Parents experience distress when uncertainty is hidden, communicated inconsistently, or treated as a reason to defer meaningful discussion. Clinicians should explain	Limacher et al. (2023)[9]; Krick et al. (2020)[26]; Boss

	what is known, what is uncertain, why uncertainty exists, what outcomes remain possible, and when greater clarity may emerge. Limacher et al. found that uncertainty sometimes delayed discussion of all available options, including palliative care.	et al. (2017)[8]
Revisit prognosis repeatedly as the infant's condition evolves	Prognostic communication should be treated as a longitudinal process rather than a one-time disclosure. Parents' understanding, emotional readiness, and preferences change over the NICU course, particularly after new diagnoses, treatment responses, complications, or prolonged uncertainty.	Arnolds et al. (2018)[19]; Krick et al. (2020)[26]; Harvey et al. (2013)[39]
Use treatment trials with explicit goals and reassessment plans when prognosis is uncertain	Families may value a time-limited opportunity to determine how an infant responds to treatment. When such an approach is used, clinicians should explain the purpose of the treatment trial, what outcomes will be monitored, when reassessment will occur, and how the results may influence subsequent recommendations. Families in Arnolds et al. often viewed the NICU experience positively even when prognosis was poor or the infant died, reflecting the importance of being given a meaningful opportunity to try treatment.	Arnolds et al. (2018)[19]; Limacher et al. (2023)[9]
Clearly distinguish prognosis sharing from decision support	Providing information about likely outcomes is not the same as supporting a treatment decision. When a decision is required, clinicians should explicitly identify the decision, describe reasonable options and their consequences, assess parental values and desired role, and offer a professional recommendation when appropriate. In Limacher et al., clinicians often described decision-making as shared, but parental preferences were not routinely elicited.	Limacher et al. (2023)[9]; Frize et al. (2013)[31]; De Proost et al. (2025)[38]
Use clear language and check understanding without testing parents	Prognostic terms, probabilities, imaging findings, and descriptions of disability may be interpreted differently by clinicians and parents. Clinicians should use plain language, pause for questions, ask parents to explain their understanding in their own words, and correct misunderstandings sensitively.	Boss et al. (2010)[30]; de Wit et al. (2013)[20]; Harvey et al. (2013)[39]
Maintain continuity and consistency across clinicians	Consistent therapeutic relationships help parents build a coherent understanding of prognosis. Changes in clinicians, contradictory messages, and underuse of interdisciplinary team members can undermine shared understanding. The care team should align key messages while openly acknowledging legitimate uncertainty or differences of opinion.	Lemmon et al. (2019)[27]; de Wit et al. (2013)[20]; Boss et al. (2017)[8]
Attend to parental emotion before delivering additional information	Parents may be unable to process complex prognostic information when overwhelmed by fear, grief, or shock. Effective communication requires recognition of emotional cues, empathic responses, appropriate	Boss et al. (2017)[8]; Kukora et al. (2025)[25]; Marçola et al.

	pacing, and opportunities to pause and revisit information.	(2020)[34]
Provide explicit assurance of continued care and non-abandonment	When discussing severe disability, treatment limitation, or palliative care, clinicians should reassure parents that the infant and family will continue to receive attentive care, symptom relief, and support regardless of the decision made. This helps prevent discussions of poor prognosis from being interpreted as withdrawal of clinical commitment.	Limacher et al. (2023)[9]; Arnolds et al. (2018)[19]; Kukora et al. (2023)[25]
Support prognosis discussions with written or visual information when helpful	Visual displays, decision-support tools, imaging explanations, and written summaries may help parents revisit complex information. These materials should support, rather than replace, individualized clinician discussion and opportunities for questions.	Frize et al. (2013)[31]; Harvey et al. (2013)[39]; Forth et al. (2024)[10]

Footnote: Evidence-derived strategies were based on findings directly reported by included studies, including observed communication practices, parent-reported experiences or preferences, feasibility findings, and comparative intervention results. These findings should not all be interpreted as evidence of effectiveness because the included studies used heterogeneous designs.
